# Online Virtual Nursing Placements: A Case Study on Placement Expansion

**DOI:** 10.1177/23779608221117392

**Published:** 2022-08-02

**Authors:** Amanda J. Wagg, Kate Morgan

**Affiliations:** 1 Anglia Ruskin University, Cambridge, UK; 2 The Learning Enterprise, HCRG Care Group, Chelmsford, UK

**Keywords:** student placements, virtual placements, virtual blended placements, student experience

## Abstract

**Introduction:**

A need to increase student placements, in a culture that is under unprecedented demand, means that practice providers and partner higher education institutions need to find new and innovative ways to ensure quality learning experiences for students. Virtual placements are just one way of providing student placements, and this study presents a case for this.

**Objective:**

This research aims to explore the student’s experience of virtual blended placements and to consider future sustainability.

**Method:**

This is a qualitative, exploratory study observing the attitudes, thoughts, and feelings of student nurse experiences of online virtual nursing placements. Key cases were identified, and two participants were asked to reflect upon their experiences of the blended virtual learning placement. Student evaluations, and virtual placement day plans, form part of the data collection, and results are presented in a theory building and comparative approach, adopting pattern matching.

**Results:**

Three themes emerged from the narratives, supported by student evaluations and day plans. Students reviewed the placement positively in that they were able to achieve their required practice proficiencies. Second, they discussed the way in which the virtual days were facilitated and that building relationships with professionals and peers while on placement was of high importance.

**Conclusion:**

This case study presents a novel and unique student experience within a 0–19 community nursing service, which through 3 days of practice placement supported by two virtually taught days, students were able to reach their practice proficiencies. This virtual placement model offers a unique way to reduce the pressures on frontline staff and enriches the students’ experiences.

## Introduction

The National Health Service (NHS) long-term plan (2019) aims to facilitate the Department of Health's intended 25% increase in nurse undergraduate places. NHS England and NHS improvements also announced that it would provide funding to Health Education England to match the number of places filled on university courses, with clinical placements from 2020 to 2021 (Maguire, 2021). This coupled with a reinstatement of nursing bursaries provides a positive effect on the number of nursing students.

However, alongside this, Brexit was seen to have a negative effect on international nursing recruitment, and COVID-19 added unprecedented demands on the nursing workforce. This raises concerns about the welfare of nurses, and the increasing numbers experiencing burnout (Maguire, 2021). Overall, an increase in student nurses, but a reduction and pressure on placement areas, has meant that practice providers offering students placements have needed to be new and innovative.

## Review of the Literature

This case study presents one provider unique and innovative approach to practice placements, only now emerging in the nursing literature, and does this from the students’ perspective. Simulated practice education is not a new concept. Manikins that simulate the deteriorating patient in a simulated ward have been used for many years to prepare students for practice (Broom et al., 2009), and more appropriately to this context, to teach public health (Schaffer et al., 2016). However, virtual placements within a practice setting are an innovation. To date, a few studies are emerging on simulated placement experiences in a practice setting, for example, a simulated placement experience relating to dietetics (Taylor et al., 2021), or a discussion on the opportunities of virtual placements in general (Donnelly, 2003), but articles into the evaluations of student's experiences of virtual placements are hard to find.

The existing literature lacks comprehensive qualitative student perspectives regarding their desired education (Song et al., 2021), so this research set out to have the student at the heart of its design.

### Virtual Placements

The research took place in a 0–19 integrated Health Visiting and School Nursing service, in the east of England, and part of a wider Child and Family Wellbeing service. Funding for 0–19 services, in the UK, transferred from the NHS to Local Government in October 2015, and the service is therefore commissioned by the local authority's public health department to deliver the Healthy Child Programme (PHE, 2021) across the county. The Healthy Child Programme is an evidence-based program of interventions including screening, developmental reviews, and information and guidance to support parenting and healthy choices and delivered by qualified health visitors and school nurses.

They began offering a 6-week blended program to preregistration student nurses. The blended program consisted of 3 days working alongside a Health Visitor or a School Nurse, and then two virtual practice days where students explored and reflected upon practice experiences. These virtual practice days were facilitated by the same clinical practitioners that they would be working within the practice setting; health visitors or school nurses working within the service. Both days in practice, and days learning virtually, were designed to meet the student’s practice proficiencies, and provide an understanding of the underpinning knowledge and theory of the practice placement. This model was designed to reduce the pressures on frontline staff, but also aimed to enhance the student's placement experience.

### Objectives

This study presents a case for blended learning placements and explores the students’ experiences of this approach. Innovations and ideas for student placements are moving quickly in practice, especially with the development of virtual placements across the country (Salmons et al., 2021). The aim of this study is to explore virtual placements, now, as they are happening, with a view of researching whether this is a viable long-term plan. This research has sustainability and student experience at the heart of its design, to inform future developments using this model. As a practice/university collaboration, this is an innovative and experimental design, that has the potential to impact future demands on student training. As part of the collaboration, the use of language was very important, emphasizing a practice experience and not further university days.

## Method

### Design

Case study is a systematic enquiry into a phenomenon and aims to describe, explain, and explore a phenomenon of interest (Bromley, 1991). A case study approach explores the how and why question, without manipulation of behavior and in the context in which it occurs (Yin, 2018). A case study that is planned and clear from the start determines the quality of the case study (Thomas, 2016), and there are many ways to explore a case (Zucker, 2001). What is central to the process is binding the case through time, place, activity, definition, and context.

### Determining the Type of Case

To ensure the quality of the case study the subject, purpose approach, and process will be clearly determined and set out to best answer the research question (Mays & Pope, 2000), and the line of enquiry planned and made clear from the start. Merriam (1988), Stake (1995), Bassey (1999), de Vaus (2001), and Yin (2018) all categorize the types of case study in different ways, but across all types, Thomas (2016) highlights that the importance throughout is the consideration for the case, purpose, approach, and process of the case design (Table 1).

**Table 1.** Elements Chosen Within for the Design of This Case Study (Thomas, 2016, p. 114).

Subject	Purpose	Approach	Process
Outlier case	Intrinsic	Test theory	Single
**Key case**	Instrumental	Build theory	**Multiple**
Local Case	Evaluative	Draw a picture	
	**Exploratory**	**Descriptive**	
	Explanatory	**Interpretative**	

In this instance, a key case approach was chosen. Students that had undertaken the virtual placement were used as a good example of the phenomenon. The purpose of the study, highlighted in the study aims was to explore the students’ experiences; thus, an exploratory design was used. Given all experiences are assumed individual and subjective the exploratory design facilitates the exploration and understanding of the students’ experiences, where outcomes are not assumed.

A descriptive and interpretative approach is taken to the data provided. The descriptive approach concentrates on describing the different aspects of the phenomenon, while the interpretative approach explores the experiences of students, thus meeting the research aims. Both allow researchers to describe the student’s accounts at the same time as learning more about what is happening and why (Thomas, 2016). Multiple cases were chosen, and two students were selected to talk about their experiences, via a convenience sample, in June 2021. Both students had an experience of the phenomenon (Zucker, 2001). As the purpose is to present this case, to other higher education institutions and placement areas, the emphasis was not on large numbers of participants but rather to provide a snapshot retrospectively. This research forms a foundation for further exploration by providing a new model of student placements.

Propositions are often created by researchers in advance of undertaking case studies, they are set off decision-making concepts or behaviors that the researchers propose as typical for a given case (Woodside, 2010). This exploratory study, like many exploratory studies, does not have sufficient knowledge on which to base any propositions so this exploration is based on the research question (Zucker, 2001).

### Data Collection

Data collection for the case study was drawn from different places (Yin, 2018): retrospective interviews with two of the 17 students who have undertaken the virtual placement, attendance records for the 17 students for the 6 weeks of attendance, plans for the virtual placement days, along with 17 student evaluations, and 11 facilitator notes written at the end of each session. The hallmark of case study research is the use of multiple sources (Baxter & Jack, 2008), as it enhances credibility (Yin, 2018).

Two interviews were carried out separately on Microsoft teams and recorded at the end of June 2021. Interviews were chosen as they explore the how and why of key events and provide insight into the student's perspectives (Yin, 2018). Video calls in this instance can provide access to hard-to-reach people and are less costly in terms of time and labor therefore more effective (McIntosh & Morse, 2015), and COVID-19 secure. Online interviews also allow flexibility of time of the participant and automatic transcription when uploaded to the secure University video system “Steam”.

### Data Analysis

The analysis focuses on the students’ experiences and does not aim to generalize the findings (Stake, 1995), using Hammersley's criteria (Denzin & Lincoln, 2013), it aims to make claims clear and utilizes Bronfenbrenner's ecological systems model (Bronfrenbrenner & Morris, 1998), to ensure the context is captured. Transcripts were hand reviewed, by both researchers for accuracy against the recording and all names, and ages, have been changed to ensure confidentiality (NMC, 2021). In the write-up of case studies, Yin (2018) suggests selecting from six unique types: linear-analytic, comparative, chronological, theory building, sequenced, and un-sequenced. Theory building and comparative approaches will be taken to these interviews. A linear-analytic structure is a standard approach for research reports starting with the research problem, literature review, methods, findings, and conclusions, and the chronological structure also aids understanding and meaning-making of events over time. The aim of the research, to explore the phenomenon, did not lend itself to a theory building approach or comparative approach of reporting, for example.

To ensure quality Hammersley's criteria was used as it provides indicators for use in case studies that focus in the quality of the case study that relates to conception, construction, and conduct of the study. This criterion was considered throughout the design of the study, and a journal of thoughts will be kept during the process of data collection to ensure the data is interpreted inductively (Denzin & Lincoln, 2013). This was then considered when liaising with the second researcher and generating themes. Denzin and Lincoln (2013) state that the investigators of the constructive paradigm are orientated to the production and reconstructed understanding of the social world. For this reason, traditional criteria of internal and external validity are replaced with terms such as trustworthiness and authenticity (Zucker, 2001). This study aims to be transparent as possible so that the findings are trustworthy and the narrative is included to show authenticity. It aims to clearly outline the question and be consistent in the use of terms.

### Ethics

A “thick disguise” was adopted to address confidentiality and anonymity in detailed cases (Gabbard, 2000). Informed consent is ensured, and the motives of the researchers are openly discussed along with the rights of the participants to withdraw at any point up to analysis (Gabbard, 2000). The student's well-being was paramount throughout the study, and the university well-being service was used a source of support should the student need it.

## Results

Seventeen preregistration student nurses undertook a virtual nursing placement within the 0–19 community nursing service, in the east of England. Placements were 6 weeks in length and the students experienced 3 days a week in practice, working alongside a health visitor or school nurse on a face-to-face practice day, and two online practice days, learning and reflecting upon practice.

Rebecca was the first student interviewed, and in her second year of study. She was a 33-year-old woman undertaking the dual registration child and mental health preregistration nursing course. Lilly was the second student, was 20 years old, and a second-year student, undertaking a 3-year child branch preregistration nursing placement.

Both students were asked to describe their experiences of the blended virtual placement, and to consider their likes and dislikes, and asked to explain why. From both interviews three main themes emerged. First, the students talked about what they felt they personally gained from their placement, and what they wanted to achieve from their practice proficiencies. Second, they reflected on the facilitation of the virtual learning days, and thirdly the relationships they built with their Practice Supervisors and Practice Assessors. These three main themes were felt to be of high importance, and important in the evaluation of the placement success. To further develop discussion around these three themes, data was also taken from the student evaluation forms collected from all students.

### Achieving Proficiencies

In this first theme, the students described how they felt the placement was able to fulfill their practice proficiencies, a practice placement requirement. Students had at least 15 proficiencies to achieve while on placement, and virtual learning days aimed to explore these from a practical viewpoint and complement practice experiences: “We have 29 proficiencies in total and yeh, we need to cover 15 in each placement. You’re never going to be able to cover all proficiencies, so having these workshops it helps you cover a lot of proficiencies” (Rebecca). No student, either interviewed or who completed the placement evaluation failed to complete their practice proficiencies.

It is in this theme that the students discussed and reflected on what they personally wanted to get from the placement, and how their own expectations were, or were not met. At first, both students felt that they did not have many expectations prior to placement and knew little about the virtual placement. Rebecca said: “when I got told I’ve got a virtual placement, I was like ‘Oh my days I’m not looking forward to this…but I actually really enjoyed the virtual days”. As the placement progressed, students felt the virtual practice days complimented the face-to-face days and provided the relevant information and underpinning knowledge. These virtual practice days allowed for reflective conversations and shared practice experiences within the group, with Lilly stating: “I think it's massively important”.

To provide clarity the plans for the virtual practice days were reviewed. These plans’ identify the theme of the day, specific to the practice placement. These days provide the underpinning practice knowledge, and opportunities to reflect on practice issues with clinical practitioners. Topics covered included community profiling, child developmental assessment tools, record keeping, the UK's National Child Measurement Programme, minor illness, child safeguarding in practice, and screening and immunizations, for example. Rebecca said: “The topics that we do are really good… more informative but not necessarily having to be out there and observe it, as sometimes like safeguarding children it's not appropriate to be out there, so actually I did find it quite helpful, because there are some of the things that you are not going to be able to see when you’re on placement and not going to be able to complete your proficiencies”. The virtual practice days increased exposure to these topics and allowed those with experiences to share and reflect upon these. This allowed for peer supervision in the development and completion of their practice proficiencies: “the virtual days do really actually compliment what we’re doing in placement because it just gives you the solid foundation of what's happening” (Rebecca).

Personally, students felt the virtual placement days were different from the university theory days as part of their modules, and thus they were able to gain new information. One student evaluation read: “I do think the information we have learnt during these virtual practice days has been very different to what we have learnt in university, with the safeguarding session there may have been a bit of an overlap but nothing major at all and I quite enjoyed going over things again to solidify the knowledge that I already had. The majority of what we have learnt in university so far has been very clinically based so seeing the community side of things has been fabulous”.

### Facilitation of the Virtual Practice Days

The students discussed at length about how they would like the days facilitated and what they enjoyed, or did not, thus making facilitation of the virtual practice days a key theme. This theme explores how the virtual practice days were facilitated by clinical practitioners (Health Visitors or School Nurses), alongside the three face-to-face practice days. This was important to the student as just under half of their time was spent on the virtual practice days.

Positively, they enjoyed the virtual days when the facilitators were prepared and confident in the theme of the day/subject topic. Both Rebecca and Lilly enjoyed the group work which involved working in smaller groups, of six or less, enabling interaction with their peers. This enabled students to have the confidence to talk and discuss the issues raised with their cameras on: “People are more likely to talk in the smaller groups. Six people is perfect really and then you can do breakout rooms as well and you can do two groups of three” (Lilly). These favorable smaller groups online also facilitated students to research topics that impact on practice for themself. One student evaluation referred to this as “doing rather than seeing”. From the practice day plans, we can see that discussion around topics such as domestic violence was facilitated. On his occasion, students were asked to consider how this law could be approached with families, looking at the softer nursing skills around effective communication, and evidencing how policy relates to practice.

Although one piece of feedback read: “I don’t personally think there is a lot that could be done to improve the virtual days”, other evaluations were more constructive, and one student wrote that they disliked sessions that consisted mainly of PowerPoint slides. Sessions with long PowerPoint slides were often described to be harder to focus on, and students reported a loss of concentration, especially if there were not frequent breaks: “I definitely think the ones where we have had more discussions about topics rather than just going through PowerPoint after PowerPoint have been more beneficial” (student evaluation).

A review of the practice day plans showed a few sessions that lent itself to a full day of facilitation with 53 PowerPoint slides. This session occurred in week four and discussed record keeping in practice. Upon reviewing the written evaluations, another student supported the need for sessions to be less PowerPoint led: “we have touched on record keeping and confidentiality at uni [university] before but not in as much depth as we have done it in our virtual practice day” (student evaluation).

Virtual practice days brought much discussion around the use of cameras. The students found being on screen hard, in that they were worried that they would be judged. There was a lack of confidence around having their cameras switched on so that other people in the meeting could see them. However, with the cameras not being on Lilly found it hard not being able to “read the room”, seeing the interactions of others. Student evaluations showed a concern that this would have an impact on building trust and therefore may prevent some students from sharing information. As the students became more comfortable, they felt it would be better to have more opportunities to have the camera on. One of the students in their end of placement written evaluation stated that she wanted: “more camera on opportunities as I feel like that makes sessions more enjoyable”.

The students liked being heard and commented that they felt the facilitators had listened to their feedback. For Lilly, the virtual practice days were too long but had felt that her voice had been heard: “I wouldn't go as far as to say I've enjoyed it because I do think it's long. It's a long day on here, but it's not as bad as it was. It's like I don't dread going onto it. I don't fall asleep when I'm on it up because I'm talking, and they give us the opportunity to talk now. And it's yeah, it's nice that they have taken on board my feedback and they've done something about it now”.

### Building Relationships

The third theme, evidenced in the narratives and evaluations, focused on whether they felt able to build relationships with their peers, Practice Assessors, or Supervisors, making this an important theme when exploring the success of virtual practice placements. Bonding and getting to know peers were recurrent themes noted: “I know it's not part of the course, but there was no like bonding exercises…the facilitators were really nice but there wasn’t that bonding at all…they [students] don’t like talking and they don’t like their cameras on” (Lilly). Similarly, one evaluation also read: “What would be better if we had more discussions throughout the day and more camera on opportunities as I feel like that makes the sessions more enjoyable seeing other faces!”.

Reviewing the practice days plans, it was evident no structured team building was incorporated into these days. However, through analysis of the facilitators daily notes it was clear that opportunities for group work, and reflection were given. One facilitator wrote: “all students put their cameras on and shared how their week had been”. Through the facilitators notes, it was apparent the journey of online virtual practice days had been a progression. At the start of the placement one facilitator wrote: “still struggling to engage the students as a group, they were quiet and do not like their cameras on”. While by the latter part of the placement students reported: “engaged well and made many contributions”.

The two interviews heard about the difficulties of studying during the pandemic: moving to a new part of the country and missing out on social activities where they get to know others. In theory, and in practice, Lilly wanted to make friends and felt the university lecturers and practice providers could have a role to play in supporting this. Students were much happier when building relationships with others and undertaking activities that supported bonding.

## Discussion

As noted in the background, this is among the first studies to provide an in-depth qualitative exploration of a blended learning approach to practice placements for preregistration nursing students. The aim of the study was to explore the experiences of students to ascertain what works well and what needs to be improved, to inform future practice. Sustainability of the blended practice placement, with the student at the heart of the experience, was the key aim.

During the introduction, the importance of expanding placements, increasing students’ numbers, and protecting the current workforce were stressed and this research provides a snapshot in time, it presents just one innovation in response to the demand and need for more student nurses. Protecting the current nursing workforce from an increase in student numbers and the burden of the pandemic is a key consideration in practice (Alameddine et al., 2021). Recent studies have reported higher levels of anxiety, emotional exhaustion, and depression among frontline workers during the COVID-19 crisis (Labrague & De Los Santos, 2020), putting them at risk of burnout (Galanis et al., 2021). This innovation is designed to increase student capacity while protecting the current workforce.

Student nurses are assessed in both theory and in practice to ensure that they are knowledgeable in their evidence-based practice and attain competence to ensure confidence in their experiences when making decisions related to patient care (Bramer, 2020). Each year of study students must demonstrate a range of clinical skills deemed essential nursing skills by the Nursing Midwifery Council (NMC, 2019). It was highlighted that the student's agenda to pass their practice proficiencies and to have a positive experience in which they grow in skills and confidence were important. It is therefore not surprising that one main theme is that students want to fulfill their proficiencies. Clinical placements have long been perceived as physical experiences in which proficiencies are undertaken, but there is a need to move away from this notion for many years now (Donnelly, 2003). This research shows that blended practice placements can support students to fulfill their practice proficiencies.

Nurse training is complex in nature, having to include professional, regulatory, theoretical, and clinical components (Smith et al., 2009). The focus of the virtual placement days was on the clinical component which the students evaluated positively in the evaluation forms and interview narratives. There was also consideration to not only proficiencies and knowledge, but the attributes required of the profession (Schnetter et al., 2014). Virtual practice days and face-to-face practice days also assessed the students against the NHS England's professional values: care, compassion, courage, commitment, communication, and competence (NHS England, 2012). These core nursing values were at the heart of the virtual placements as students explored the practical application of policy, practice, and theoretical practice components to ensure they could deliver safe, effective communication and care to the families in the 0–19 children's nursing service.

Student experiences are a key performance indicator for the UK universities (Department of business innovation and skills, 2016) and the emphasis on student satisfaction. This research goes some way in evidencing views on this blended virtual placement, and how the students liked the days to be facilitated. Shorter PowerPoints and regular group work that increases peer interaction were the main points to emerge from the data. Literature on Midwife education has also shown that active learning over lecture-only sessions support improves test scores and is very acceptable to students (Everly, 2013), and a dislike of text-heavy PowerPoints was common (Shelton, 2018). The sub-theme around peer support was raised on numerous occasions both in interview and student evaluations, and an important theme in the nursing literature. Loss of interaction with peers and feeling lonely were considered disadvantages of online learning, whereas a full practice experience would involve increased communication and contact with others through collaboration (Banna et al., 2015). Therefore, the findings of this study, and the findings in the literature, is the need to allow time for collaboration and peer communication, which also provides a social support (Bandura, 1977).

Lastly, this research sheds light on the importance of the relationships built while on placement. Clinical practice experiences have the greatest influence on student’s desire to continue their studies and negative experiences are linked to attrition and stress (Crombie et al., 2013; Hamshire et al., 2013). Students require positive placement experiences, facilitated by effective practice supervision (Jack et al., 2018). Research shows that not all students have positive experiences conducive of learning, some placements result in loss of self-esteem and authentic self and a source of stress (Randle, 2002).

Good placements have been described as those that provide a sense of belongingess (Levett-Jones & Lathlean, 2008) and supportive relationships with mentors/practice supervisors are essential (Crombie et al., 2013), and often the most important impact on the learners and their development (Jack et al., 2018). Smith and Gray (2001) have suggested matching student nurses with specific supervisors, considering their unique and individual interests and personalities. Rather than a “one size fits all” approach to student supervision, there can be an encouragement of relationship building between certain individuals, not only between mentor and student, but involving the whole team.

Overall, lessons have been learnt and continue to be learnt as this innovation constantly morphs and improves, as highlighted above. This research goes part way in describing one innovation, as a consideration to other practice providers, as quality is a priority for all involved in higher education (HEA, 2014).

### Study Strengths and Limitations

Case study approach has often been criticized for lacking scientific rigor and providing little basis for generalization (Yin, 2018). Case study is also dependent on the wider political and social environment. These results present two cases, and although these narratives provide key themes these should not be over generalized. Alternative explanations around the experiences of other students undertaking this placement are possible. However, case study design does present flexibility to collect data through various means and capture the context and lived reality of participants that provides valuable insight.

## Conclusion

This research presents a case for a new and innovative practice placement experience for student nurses. This case study presents a novel and unique student experience within a 0–19 community nursing service, in which through 3 days of practice placement supported by two virtually taught days, students were able to reach their practice proficiencies. This virtual placement model, based in the east of England, offers a unique way to reduce the pressures on frontline staff, and enrich the students’ knowledge and understanding of professional and practical nursing care for children, young people, and families.

### Implications for Future Practice

With sustainability at the heart of the design, along with the experiences and needs of the students, this blended learning approach with virtually taught elements proves successful in supporting student nurses in clinical community practice. Although firm conclusions cannot be drawn from a single case study design, this case demonstrates a new direction for student nurse training, and the virtual practice placement model is a sustainable and effective model for future use.
